# Effects of Build Orientation and Loading Direction on the Compressive Behavior of Additively Manufactured Re-Entrant Auxetic Materials

**DOI:** 10.3390/polym17233123

**Published:** 2025-11-25

**Authors:** Mehmet Ermurat, Mevlut Safa Dag

**Affiliations:** Department of Mechanical Engineering, Kahramanmaras Sutcu Imam University, Kahramanmaras 46050, Türkiye; mevlutsafadag@hotmail.com

**Keywords:** re-entrant honeycomb, auxetic materials, additive manufacturing, fused deposition modeling, digital light processing, building orientation

## Abstract

Additive manufacturing (AM) technologies, particularly Fused Deposition Modeling (FDM) and Digital Light Processing (DLP), offer viable solutions for producing Auxetic materials characterized by their negative Poisson’s ratio. This study investigates the influence of build orientation and loading direction on the mechanical behavior of re-entrant honeycomb auxetic structures fabricated using both FDM- and LCD-based DLP techniques. Specimens were produced in three principal build orientations (X, Y, and Z) and subjected to compression along two directions (X and Y) to capture the anisotropic mechanical response. Standard PLA filament was used for FDM, while standard and tough resins were used for DLP. Uniaxial compression tests were conducted to evaluate maximum compressive stress, Poisson’s ratio, and energy absorption behavior. The results reveal significant anisotropy in mechanical performance depending on build orientation and printing technology. DLP-printed specimens exhibited more isotropic behavior compared to FDM due to superior interlayer adhesion. Furthermore, build orientation was found to have a pronounced effect on auxetic response and load-bearing capacity. This study highlights the critical importance of considering build orientation and loading direction during the design and manufacturing of auxetic structures, especially for applications requiring targeted mechanical performance.

## 1. Introduction

Conventional materials exhibit the Poisson effect, meaning they contract laterally when subjected to tensile forces and expand when compressed. In contrast, auxetic materials possess a negative Poisson’s ratio, expanding laterally when stretched and contracting when compressed [1,2]. Auxetic materials have high properties in terms of energy absorption capacity, fracture toughness, notch resistance and shear resistance and are differ from traditional materials with the same chemical properties [3].

**Figure 1:**
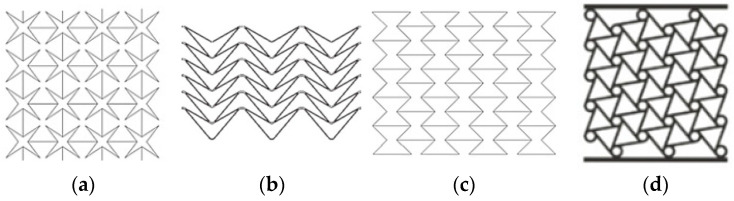
Some examples of non-natural auxetic structures (**a**) Star shaped [4], (**b**) Arrow Shaped [5], (**c**) Re-entrant honeycomb [4], and (**d**) Chiral [6].

Auxetic behavior can be found in both natural systems, such as cancellous bone and zeolites, and in engineered architectures inspired by these structures. In recent years, numerous geometric models demonstrating auxetic behavior have been investigated and experimentally characterized. Representative designs commonly cited in the literature are shown in Figure 1.

The complexity of auxetic structures—particularly the requirement for regular pores or cellular patterns—makes their fabrication with conventional methods challenging and costly [7]. However, current technologies—particularly 3D printers—allow for the easy manufacturing of auxetic materials at various scales and with complex lattice structures. This opportunity, combined with current engineering trends such as the multifunctionality of auxetic structures for shock absorption, as well as for sensors or energy storage, has fueled development. The capability of Additive Manufacturing (AM) to produce such complex geometries with high precision is therefore essential for advancing auxetic applications.

AM, also referred to as layered manufacturing, fabricates components by sequentially depositing material in a layer-by-layer manner onto a build platform [8]. AM technologies are increasingly adopted across various industries—including aerospace, defense, automotive, medical, and tooling sectors—due to their capability to fabricate geometrically complex parts with high precision, shorter lead times, and enhanced mechanical performance [9,10,11,12,13,14,15,16].

Anisotropy in AM parts arises from their layer-by-layer fabrication, where interlayer bonding is typically weaker than intralayer cohesion [17,18], leading to superior mechanical properties in the XY plane compared to Z-axis. This variation in adhesion and material distribution, influenced by build orientation, differs depending on the specific AM technology used [19,20].

This phenomenon is particularly evident in Fused Deposition Modeling (FDM), where layer adhesion is weak compared to in-plane bonding, resulting in lower strength along the Z-axis. Digital Light Processing (DLP), on the other hand, offers improved interlayer adhesion due to chemical curing, rendering more isotropic behavior when optimal curing conditions are achieved. The layers are chemically bonded and cured in a single step, resulting in stronger interlayer adhesion compared to FDM. Mechanical properties in DLP printed parts are therefore more isotropic than in FDM parts.

Lattice structures with periodic voids are difficult to fabricate using traditional methods [21], but AM enables their precise and customizable production, which is especially beneficial for auxetic designs with complex geometries. Due to their high strength-to-weight ratio, such structures are efficiently manufactured via AM and widely applied in industries like automotive, aerospace, defense, and medicine, with ongoing developments in lattice topologies and design software [22,23,24,25,26,27].

Certain auxetic geometries with intricate lattice features can only be fabricated using AM, making it not just advantageous but sometimes the only feasible fabrication method. Owing to their wide elastic deformation range, polymers are commonly used in AM of auxetic materials; while FDM is the most prevalent technique, studies employing DLP or SLA remain limited [23,28].

Weak interlayer bonding and the staircase effect, commonly observed in many AM processes, have been reported to reduce the strength of auxetic materials [29]. Therefore, build orientation, printing method, and material properties play a crucial role in determining the mechanical behavior of auxetic structures. Changing the printing orientation alters the anisotropic characteristics of the part, which in turn affects the mechanical properties of auxetic materials in a similar way to conventional parts [30]. By selecting an appropriate build orientation for the chosen AM technique, anisotropic effects can be controlled to achieve optimal auxetic performance, tailored for desired strength, energy absorption, or both, depending on the application.

On the other hand, another consideration in AM is the necessity of creating a support structure in some cases, depending on the build orientation and part geometry. The formation and subsequent removal of these supports increased production time and cost while introducing surface defects such as cracks, notches, or roughness. Additionally, the surface irregularities caused by both support removal and the staircase effect act as stress concentrators, leading to premature crack initiation and reduced mechanical strength [31,32,33]. These issues are particularly critical for porous or hollow auxetic structures, where surface imperfections can significantly weaken the material.

Therefore, when designing an auxetic material to be manufactured with additive manufacturing, the build orientation and the direction of the forces the part will be subjected to should be considered [34]. Although some studies on different building orientations have been encountered [35,36,37], there are no comprehensive studies in the literature that systematically compare the combined effects of both build orientation and loading direction on the material performance of auxetic structures fabricated with AM for different AM technologies (especially FDM and DLP/LCD) and polymer types. This gap complicates the selection of the most appropriate fabrication parameters and technology for specific applications.

To address the gap in existing research regarding the effects of build orientation and loading direction on the mechanical performance of auxetic structures, this study comprehensively evaluates the behavior—specifically maximum compressive stress, Poisson’s ratio, and energy absorption—of a re-entrant honeycomb auxetic structure fabricated using three principal build orientations (X, Y, and Z axes). The primary goal is to address the current literature gap by providing a systematic, multi-parameter analysis. Compression tests were conducted in two loading directions (X′ and Y′), as the Z-axis loading was excluded due to the absence of auxetic response in that direction. The specimens were manufactured using two different additive manufacturing techniques: FDM with standard PLA material, and LCD-based DLP (LCD-DLP) with both standard and tough resin types, allowing for a direct comparison of process-induced anisotropy. The mechanical performance of the specimens was characterized through uniaxial compression testing.

These tests enabled a comparative analysis of material behavior as a function of build orientation, loading direction, and manufacturing method, thus contributing to a deeper understanding of design considerations for auxetic applications. Specifically, the findings will establish critical design guidelines for selecting the optimal AM method and build orientation to maximize targeted auxetic performance characteristics (e.g., maximizing energy absorption or achieving the lowest Poisson’s ratio) based on the end-use application.

This experimental study has some limitations due to its scope: it is limited to a re-entrant honeycomb structure exhibiting biaxial (planar) auxetic behavior with a fixed geometry and size; and the performance of different cell geometries and sizes or three-dimensional auxetic structures is not investigated. The material selection is limited to polymer-based materials (PLA for FDM, Standard and Tough Resin for LCD-DLP). Furthermore, mechanical characterization is performed only through uniaxial static compression tests, and complex loading conditions such as dynamic, impact, or fatigue, which are critical for the energy dissipation potential of auxetic materials, are excluded from the scope of the study.

## 2. Materials and Methods

### 2.1. Specimen Design and Experimental Plan

**Figure 2:**
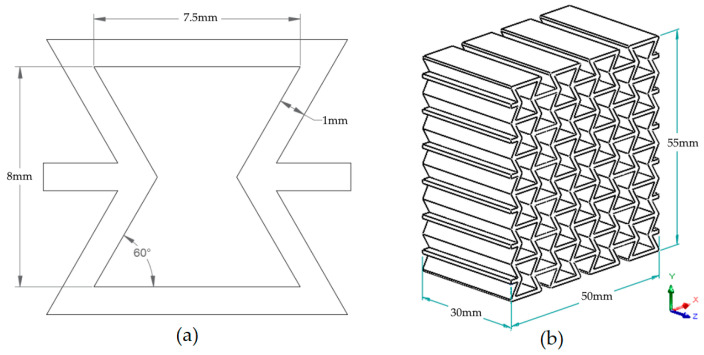
(**a**) Re-entrant cell structure dimensions (**b**) Re-entrant honeycomb specimen geometry and dimensions.

In this study, the re-entrant honeycomb structure was selected as the auxetic model due to its frequent use and proven performance in the literature. The dimensions of a single re-entrant cell, as illustrated in Figure 2a, were used to construct the overall geometry. By arranging these cells in rows and columns in a repeating pattern, a lattice-based specimen was designed, as shown in Figure 2b.

Re-entrant structures exhibit anisotropic behavior in nature [38], and accurate evaluation in experimental studies requires accounting for these directional properties. Therefore, compression tests must be conducted along each principal direction to fully characterize their mechanical response [35]. For each build orientation, two groups of specimens were fabricated to enable compression testing in two loading directions (X′ and Y′), resulting in a total of six specimens per material type.

To investigate the anisotropic mechanical behavior of the structures, specimens were printed in three principal build orientations (X, Y, and Z). Support structures were required for printing in the X and Y orientations, whereas the Z-oriented specimens were printed without supports due to the self-supporting nature of the geometry in that direction. This setup constituted a full factorial experimental design examining the effects of three independent variables: material type (PLA, Standard Resin, Tough Resin), build orientation (X, Y, Z), and loading direction (X′, Y′). A total of 3 × 3 × 2 = 18 specimen groups were prepared, with one compression test conducted for each unique configuration.

### 2.2. Three-Dimensional Printing Apparatus and Materials

Two types of 3D printers were employed in this study, both manufactured by Creality 3D (Shenzhen, China): FDM Creality Ender3 V2 and LCD-LCD Halot One. The materials used were Porima PLA filament for FDM, and Creality 3D Standard Resin and Anycubic Tough Resin for LCD-DLP. PLA filament material is generally used for education, low-cost rapid prototyping, and static end-user products; Standard Resin is preferred in precision applications requiring high resolution and detailed models, and Tough Resin is preferred in engineering and functional prototypes requiring high mechanical strength and impact resistance.

Post-processing procedures varied by printing method. For the FDM-printed specimens, support structures were manually removed. In the case of the LCD-DLP-printed specimens, support removal was followed by washing in isopropyl alcohol to remove residual uncured resin. Specimens were then post-cured under uniform conditions (10 min) using the Creality 3D (Shenzhen, China) manufactured Creality UW-01 curing station. All printing and post-curing parameters were selected based on the technical specifications recommended by the respective printer and material manufacturers. Specific printing parameters for both FDM and LCD-DLP processes are presented in Table 1 and Table 2, respectively.

### 2.3. Mechanical Testing Setup and Procedure

Compression testing was conducted to evaluate the mechanical response of the auxetic specimens, as the design was optimized for compressive loading. Tests were carried out along the X and Y axes; testing along the Z-axis was omitted since the auxetic response is not expected in this direction due to the geometric characteristics of the structure.

After post-processing and surface cleaning, the printed specimens were positioned between the plates of Zwick/Roell universal testing equipment (ZwickRoell GmbH & Co. KG, Ulm, Germany) and tested at a constant compression rate of 5 mm/min until permanent deformation or collapse occurred. For each material group, the previously printed six specimens—two for each build orientation (X, Y, and Z)—were tested such that one specimen per orientation was loaded along the X′ direction and the other along the Y′ direction, with each specimen tested only once.

The tests were recorded using both a standard video camera and a digital microscope that focused on selected cells, capturing specific frames for detailed observation. The tests were recorded using both a standard video camera and a digital microscope that focused on selected cells, capturing specific frames for detailed observation. The video recordings were used to calculate Poisson’s ratio and to support the evaluation of deformation kinematics between cells, while the microscope images enabled detailed examination of cell-level deformation patterns at different stages of compression.

**Figure 3:**
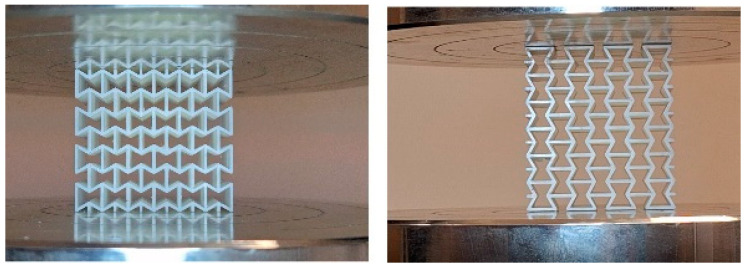
Placement of the specimens on the directions of X′ (**left**) and Y′ (**right**) on the compression test apparatus plate.

Figure 3 shows the test setup, including the placement of specimens between the compression test apparatus plates in both X′ and Y′ directions. Orientation of the applied loading directions can be seen in Figure 2b.

The testing configurations are also summarized in Table 3, which introduces a naming convention for each specimen type according to the re-entrant honeycomb geometry entitled in Figure 2b.

In this notation, the first character represents the build orientation, and the second character denotes the loading direction of compression. In the schematic representation column, red lines and planes show the layering phenomena and the arrows show the compression direction.

For stress calculations, the critical cross-sectional area—defined as the smallest projected area along the loading direction—was used.

(1) $$\sigma_{max}=F_{max}/A_{critical}$$

**Figure 4:**
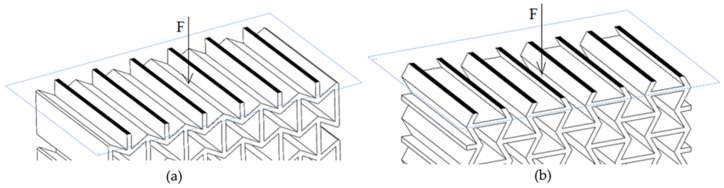
Critical cross-sectional area at loading direction of (**a**) X (X′) and (**b**) Y (Y′).

The maximum applied force (F_max_) was divided by this area (A_critical_) to obtain the Maximum Compressive Stress (σ_max_) value, as described in Equation (1). These critical cross-sections were identified by analyzing 3D CAD models and are visually marked in black in Figure 4 for the X and Y orientations.

The toughness of each specimen was determined by calculating the area under the stress–strain curve using the trapezoidal rule, as presented in Equation (2) [39,40].

(2) $$U_{t}\approx \sum_{i=1}^{n-1}\left(\left(\sigma_{i}+\sigma_{i+1}\right)/2\right)*\left(\varepsilon_{i+1}-\varepsilon_{i}\right)$$

The Specific Absorbed Energy (SAE) was calculated as the ratio of toughness to the density of the specimen, as in Equation (3).

(3) SAE=Utρ

Deformations in both longitudinal and transverse directions were quantified using Image 1.54p J software by comparing the initial and deformed dimensions extracted from the video frames captured at the beginning and during the compression tests. Poisson’s ratio was then calculated using Equation (4).

(4) v=−εy/εx=−(∆dd0)/(∆ll0)

## 3. Results and Discussion

**Figure 5:**
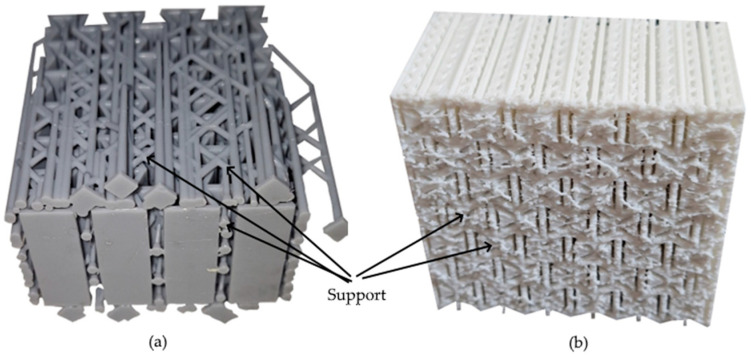
Support structures of specimens (**a**) Standard Epoxy built in Y orientation (**b**) FDM-PLA built in X orientation.

**Figure 6:**
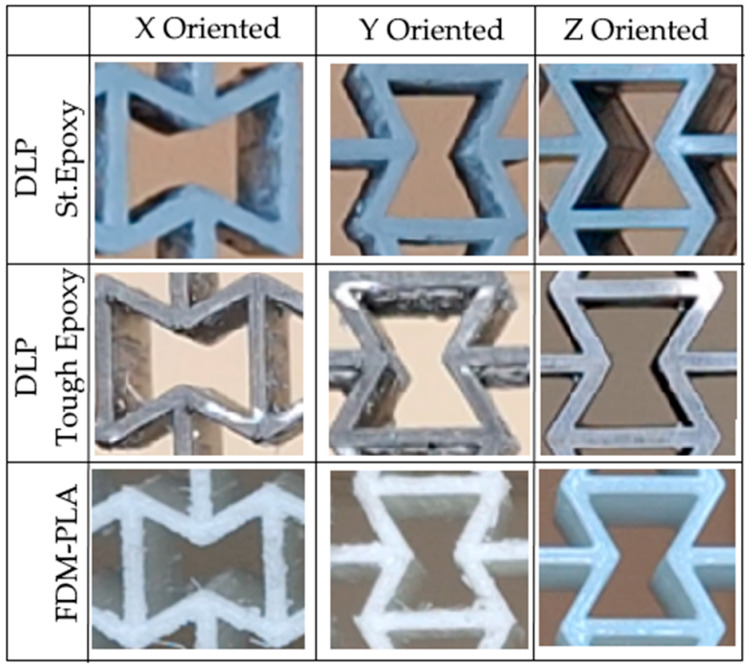
Visualizations of an auxetic cell of specimens after support removal.

Regardless of 3D printing method and material, specimens produced in the Z direction had smoother surfaces due to the lack of a support structure, while specimens produced in the X and Y directions had rougher surfaces, particularly on the surfaces where the supports were attached. Figure 5 shows examples of some specimens with support structures. Figure 6 displays the visual appearance of all specimens following post-processing, highlighting the surface defects remaining on the X and Y oriented specimens due to support removal, and the comparatively smooth finish achieved on the Z-oriented specimens.

**Figure 7:**
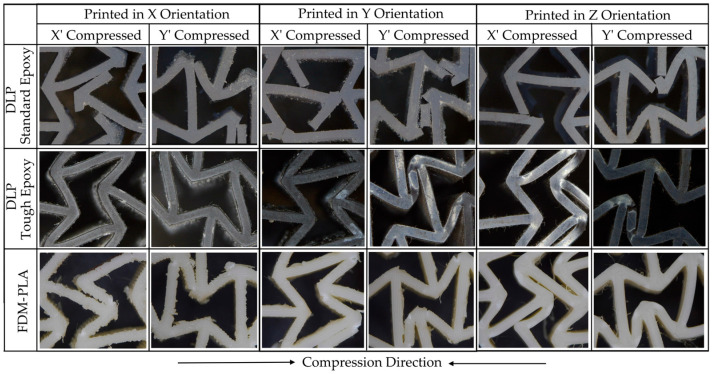
Microscopy visualizations of the auxetic cells after compression tests.

Figure 7 presents captured micrographs of the specimens after failure or when the compression test was terminated. These images show different auxetic cells at various stages of the experiment; they do not represent specimens subjected to the same compression rate.

**Figure 8:**
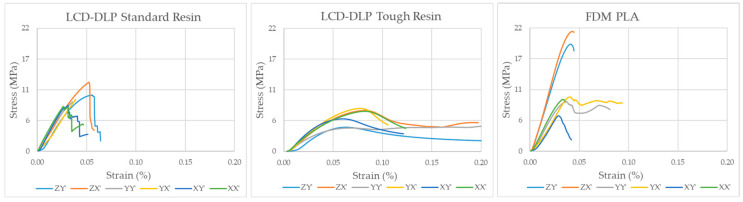
Stress–Strain Diagrams.

Figure 8 shows the stress–strain responses of the auxetic specimens, plotted on identical scales to enable direct comparison of their mechanical behavior under compression.

Figure 7 and Figure 8 collectively illustrate the distinct mechanical responses and failure mechanisms of the auxetic specimens produced by different additive manufacturing methods. The LCD-DLP Standard Resin exhibited brittle behavior with abrupt stress drops and limited deformation, accompanied by fracture-type failures observed in the micrographs. In contrast, the Tough Resin showed a ductile and stable response, maintaining structural integrity without fracture and demonstrating near-isotropic performance. The FDM PLA specimens reached the highest strength levels but displayed strong anisotropy, where X′ and Y′ compressed specimens failed through layer delamination and separation, while Z′-compressed ones exhibited only partial delamination with delayed fracture due to the load alignment with the deposition layers.

**Figure 9:**
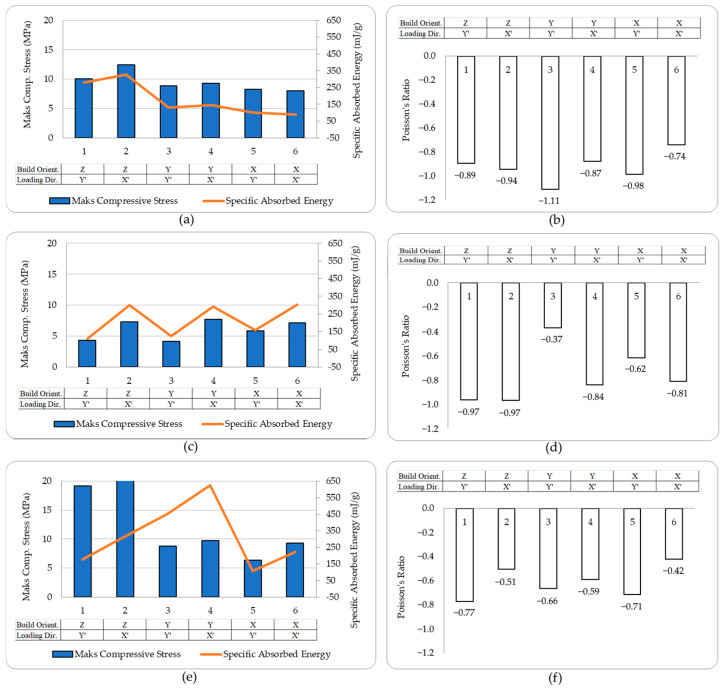
Maximum Compressive Stress and Specific Absorbed Energy (**a**) Standard Epoxy, (**c**) Tough Epoxy (**e**) PLA and Poisson’s Ratio (**b**) Standard Epoxy, (**d**) Tough Epoxy (**f**) PLA Results.

All experimental results were calculated using Equation (1) through Equation (4) and are presented in the graphical format shown in Figure 9. In these graphics, *σ_max_* and *SAE* values are presented on the left, while *ν* results are illustrated on the right.

The mechanical responses of the specimens, determined by their building orientations and loading directions in the compression tests, were evaluated with respect to the 3D printing method and material.

### 3.1. LCD-DLP—Standard Epoxy

The LCD-DLP printed standard epoxy specimens revealed limited variation in σ_max_ across different orientations (typically 8–10 MPa), with ZX′ specimen reaching 12.5 MPa. However, a much stronger orientation effect was observed in SAE, where Z-oriented specimens, especially X′-compressed (ZX′) exhibited up to 330 mJ/g, more than twice the SAE of other configurations. This enhancement is attributed to aligned stress paths along the print layers, which may improve load transfer under axial compression and reduce premature delamination [41]. This highlights the critical role of printing orientation in optimizing SAE, as Z-oriented printing likely promotes favorable layer bonding or cellular geometry that enhances energy absorption under X′-direction loading, crucial for applications such as protective gear or crash structures due to requiring impact resistance where auxetic materials structures.

The most favorable auxetic behavior was observed in YY′ specimens, which reached ʋ of −1.1, outperforming others (typically −0.7 to −0.95). This supports the observation that in-plane alignment of load and print directions promotes more cooperative lateral expansion. Such strong auxetic performance makes the YY′ configuration a promising candidate for applications requiring large lateral expansion under axial load, such as biomedical stents, wearable devices, morphing structures, soft robotics and adaptive seals where dimensional adaptability and expansion-induced functionality are critical, making it particularly suited for applications requiring flexibility, conformability, or controlled deformation [42].

Regarding the failure mechanism, it was observed that for all printing and loading directions, failure occurred nearly independent of printing orientation, indicating that the LCD-DLP method produces an isotropic structure that maintains this characteristic even in fine cellular geometries. However, specimens printed in the Z orientation exhibited slightly delayed fracture compared to those printed in other orientations, particularly those compressed in the X′ direction. The LCD-DLP Standard Resin specimens showed a brittle response, characterized by a sharp rise and sudden stress drop, indicating rapid crack propagation and limited deformation capability. Minor variations in strength were observed among orientations, with specimens printed in the Z′ orientation exhibiting delayed failure, likely due to smoother surface formation and more uniform layer bonding. Overall, the results highlight the impact of surface irregularities and support removal processes, which introduce stress concentrations and diminish the mechanical performance of the printed structures which was referenced in [29,30,31].

### 3.2. LCD-DLP—Tough Epoxy

In the tough epoxy group, the compression direction dominated performance, with X′-compressed specimens achieving higher σ_max_ and SAE values regardless of print orientation. This supports the idea that load alignment across layer planes enhances structural confinement and strain energy transfer, a mechanism emphasized in curved-rod-based auxetic unit designs [41].

X′-compressed specimens consistently showed higher σ_max_ (7.5 MPa) and SAE (300 mJ/g) than their Y′-compressed counterparts (5 MPa, 120 mJ/g), confirming the importance of load direction over print orientation in ductile materials. Hence, the build orientation of tough epoxy had a negligible influence on σ_max_ and SAE, confirming an almost isotropic mechanical response driven mainly by the material’s inherent ductility rather than print anisotropy. Additionally, from a structural perspective, Tough resin is inherently a flexible material and has lower strength. As a result, its ability to develop a reaction force against the applied compression load was relatively low.

A notable exception occurred in ZY′ specimens, which displayed the highest auxetic response of (ν = −0.98) among Y′-loaded specimens. This deviation from the average (ν ≈ −0.5) likely results from torsion-dominated deformation being confined and stabilized by vertically aligned layers, leading to more symmetric lateral expansion [43].

**Figure 10:**
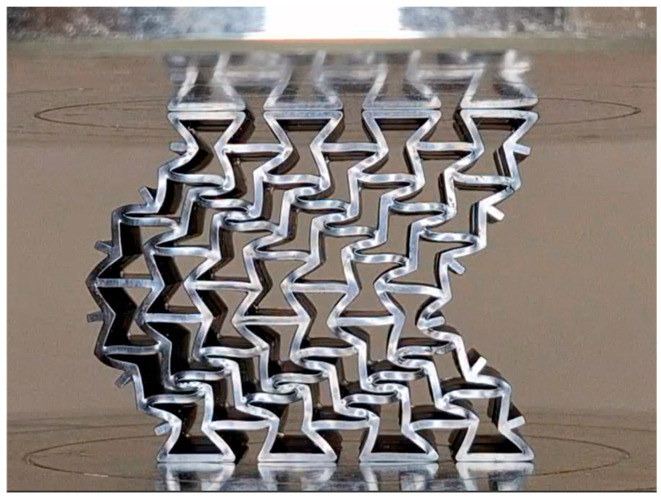
Bending sideways of the tough material during compression test in Y′ direction.

Reduced mechanical behavior under Y′-compression may be associated with bending sideways in early stages, as illustrated in Figure 10.

Unlike the brittle failure observed in standard epoxy and PLA specimens, tough epoxy specimens withstood compression without fracturing (Figure 7), demonstrating superior ductility and structural resilience. The absence of fracture in all tough epoxy specimens demonstrates this material’s improved ability to undergo large deformations and delay catastrophic failure, which is crucial for resilient auxetic systems in dynamic loading environments.

No fracture was observed in the specimens built with Tough Resin, which demonstrated a significantly more ductile response compared to Standard Resin. The stress–strain curves exhibited a smoother profile with gradual stress reduction after the peak, indicating controlled deformation and improved energy absorption. Although σ_max_ values were slightly lower than those of the Standard Resin, the higher deformation capacity and minimal orientation-dependent variation suggest that LCD-DLP Tough Resin exhibits nearly isotropic mechanical behavior, maintaining structural integrity even in thin-walled auxetic cells.

These findings align with previous studies [44], emphasizing that ductile polymer matrices, when combined with auxetic geometries, enable superior energy dissipation and delay catastrophic failure under dynamic loading.

### 3.3. FDM—PLA Filament

The PLA specimens manufactured via FDM showed strong directional dependence in both σ_max_ and SAE. Z-oriented specimens exhibited significantly better σ_max_ performance (averaging 20 MPa), which was more than double that of specimens printed in other orientations (averaging 8 MPa) due to continuous filament paths aligned with the loading direction, which increases interlayer cohesion [45]. This difference is believed to arise from the stair-stepping effect inherent in layer formation on inclined surfaces. In addition, the weaker interlayer bonding, especially in thin-walled regions, contributes to early failure through separation or delamination under compressive loads in X′ and Y-oriented specimens. This effect was more pronounced in specimens with smaller cell sizes, where the removal of rigid support structures frequently caused mechanical damage.

The post-processing step, particularly the support removal process, introduced additional stress concentrators in the form of surface notches, further compromising structural integrity. In addition to the difficulty of cleaning the support structures formed within the small auxetic cells [46], these notches acted as potential crack initiation sites, adversely affecting the mechanical performance. Moreover, due to layer discontinuities resulting from the staircase effect, the specimens printed in the X and Y directions exhibited fractures that initiated with interlayer separation, progressed along the layer interfaces, and ultimately led to complete specimen failure [29,31]. This phenomenon can be seen at the X and Y printed specimens in Figure 7.

Compared to LCD-DLP specimens, the FDM PLA specimens demonstrated greater structural stiffness and robustness in the Z direction, possibly due to their thicker support walls and more continuous deposition paths. Nevertheless, the observed anisotropy highlights the critical role of build orientation and support strategy in determining the mechanical performance of printed lattice structures.

In contrast to the trends observed for σ_max_, SAE values of FDM PLA specimens demonstrated a markedly different pattern. Notably, Y-oriented specimens exhibited significantly higher SAE, ranging between 450 and 650 mJ/g, which is nearly double that of the Z-oriented specimens (225–325 mJ/g).

This seemingly paradoxical behavior can be attributed to a compliance driven energy dissipation mechanism. The increased stiffness of the Z-oriented structures restricts their plastic deformation capacity, thereby limiting their energy absorption potential despite higher peak stress levels. Conversely, the relatively more flexible Y-oriented structures exhibit enhanced lateral deformation and delayed buckling, which contribute to more effective energy dissipation throughout the compression cycle.

This observed trade-off between strength and energy absorption aligns with findings from re-entrant auxetic honeycomb architectures, where energy dissipation is optimized not through axial stiffness, but through geometric compliance and deformation adaptability. Moreover, among the specimens printed in the same orientation, X′-compressed specimens absorbed approximately 1.5 times more energy than their Y′-compressed counterparts, highlighting the additional influence of loading direction on energy absorption behavior.

The FDM PLA specimens exhibited the highest peak stress values, reaching up to approximately 20 MPa. However, the stress–strain curves show pronounced fluctuations and abrupt drops, characteristic of layer delamination and anisotropic failure. Specimens printed in the X and Y orientations experienced fracture initiation and propagation along the layer interfaces, resulting in premature failure. Conversely, those printed in the Z orientation did not fracture completely but showed partial delamination, indicating delayed failure due to load alignment with the deposition layers.

These findings underline the fact that the energy absorption capacity of PLA structures produced via FDM is not solely governed by their maximum stress levels but is significantly influenced by their build orientation and deformation characteristics. Especially in Y-oriented specimens, the capacity for sustained deformation without catastrophic collapse enables continued energy absorption beyond peak stress, making them favorable for applications requiring impact mitigation or damping.

Beyond energy absorption, the auxetic behavior also showed orientation dependent variations. Y′-compressed specimens generally showed a better auxetic response (−0.65 to −0.78) than X′-compressed ones (−0.42 to −0.59), while the ZY′ configuration demonstrated the highest Poisson’s ratio among all PLA specimens.

## 4. Conclusions

This study systematically examined how build orientation and loading direction influence the mechanical and auxetic responses of re-entrant honeycomb structures, while comparing these effects across various additive manufacturing techniques and material types. The maximum compressive strength-, specific absorbed energy-, and Poisson’s ratio-based auxetic performances were influenced by the build orientation and compression direction, showing noticeable variations among different additive manufacturing methods and materials.

The loading direction emerged as the predominant factor influencing both strength and auxetic response, largely independent of the manufacturing method. Among all configurations, specimens built in the Z orientation and compressed in the X′ direction exhibited the most durable behavior. This is attributed to the absence of lateral collapse during X′ loading and the homogeneous internal structure obtained through Z-oriented fabrication, which minimizes support induced defects and stair-case effects.

Interestingly, contrary to the common trend reported in the literature [47,48], loading in the X′ direction yielded superior results compared to Y′. This deviation can be attributed to increased lateral deflection and torsional instability during Y′ loading, which induced premature local buckling and energy loss.

Anisotropic behavior was more pronounced in FDM specimens, as also referred to in [49], while DLP specimens demonstrated a predominantly isotropic response. Consequently, the build orientation is particularly crucial in the FDM method for controlling both the mechanical properties and the resulting auxetic behavior.

The complex internal cavities of re-entrant honeycomb structures made support removal a labor-intensive and damage-prone step, often introducing surface irregularities that further influenced the mechanical response for both printing methods. Consequently, some studies adopted a Z-axis (upright) printing orientation to eliminate the need for internal supports and ensure a more reliable examination of the auxetic structures [50].

Among all tested materials, Tough Resin exhibited the most stable and energy-absorbing response, while Standard Resin showed brittle fracture with sudden load drops after peak stress. FDM-PLA displayed higher strength but was limited by pronounced anisotropy and delamination-induced failure. Collectively, these findings confirm that the mechanical performance of auxetic structures is governed by build orientation, layer continuity, and material type, which together dictate the onset of failure and the efficiency of energy dissipation.

Future work should focus on microstructural characterization of the printed layers to better understand the origin of isotropic versus anisotropic behavior in different printing methods. Investigation of dynamic or impact loading conditions would provide deeper insights into the real-world energy absorption capabilities of these auxetic structures. Incorporating finite element simulations could enable predictive optimization of layer orientation and cellular topology for tailored mechanical performance.

The findings of this study suggest that for two-dimensional auxetic structures, fabrication along the Z-axis can be achieved without the need for support structures, making it the most efficient and defect-free manufacturing approach. However, in the case of three-dimensional auxetic structures, the use of support structures becomes largely inevitable. Therefore, this study provides a foundational framework for future research focused on the fabrication and optimization of fully three-dimensional auxetic materials.

## Figures and Tables

**Table 1:** FDM printing settings.

Parameters	PLA Filament
Layer height (mm)	0.12
Line width (mm)	0.4
Printing (nozzle) temperature (°C)	210
Build plate temperature (°C)	60
Infill density	100%
Print speed (mm/s)	50
Support Overhang Angle (°)	59

**Table 2:** LCD-DLP printing settings.

Parameters	Standard Resin	Tough Resin
Layer height (mm)	0.05	0.05
Bottom layer exposure time (s)	40	50
Exposure time (s)	3.5	4.5
Build platform lift distance (mm)	5	7
Build platform motor speed (mm/s)	3	3
Delay time (s)	2	3

**Table 3:** Printing orientation and loading directions, including schematic representation of each test number.

Test Type Name	Printing Orientation	Loading Direction	Schematic Representation of Testing According to Building Orientation
ZY′	Z	Y′	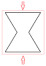
ZX′	Z	X′	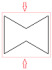
YY′	Y	Y′	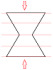
YX′	Y	X′	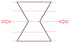
XY′	X	Y′	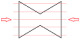
XX′	X	X′	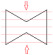

## Data Availability

The data presented in this study are available on request from the corresponding author.

## Abbreviations

The following abbreviations are used in this manuscript:

AM	Additive Manufacturing
FDM	Fused Deposition Modeling
DLP	Digital Light Processing
SLA	Stereolithography Apparatus
SAE	Specific Absorbed Energy
